# Attitudes and Behaviours to Antimicrobial Prescribing following Introduction of a Smartphone App

**DOI:** 10.1371/journal.pone.0154202

**Published:** 2016-04-25

**Authors:** Preet Panesar, Alisdair Jones, Alicia Aldous, Katharina Kranzer, Eamus Halpin, Helen Fifer, Bruce Macrae, Carmel Curtis, Gabriele Pollara

**Affiliations:** 1 Department of Pharmacy, University College London Hospitals, London, United Kingdom; 2 Department of Infectious Disease Epidemiology, London School of Hygiene & Tropical Medicine, London, United Kingdom; 3 Department of Clinical Microbiology, University College London Hospitals, London, United Kingdom; 4 Horizon Strategic Partners, Leeds, United Kingdom; 5 Division of Infection & Immunity, University College London, London, United Kingdom; University of Ottawa, CANADA

## Abstract

**Objectives:**

Our hospital replaced the format for delivering portable antimicrobial prescribing guidance from a paper-based pocket guide to a smartphone application (app). We used this opportunity to assess the relationship between its use and the attitudes and behaviours of antimicrobial prescribers.

**Methods:**

We used 2 structured cross-sectional questionnaires issued just prior to and 3 months following the launch of the smartphone app. Ordinal Likert scale responses to both frequencies of use and agreement statements permitted quantitative assessment of the relationship between variables.

**Results:**

The smartphone app was used more frequently than the pocket guide it replaced (p < 0.01), and its increased use was associated with sentiments that the app was useful, easy to navigate and its content relevant. Users who used the app more frequently were more likely to agree that the app encouraged them to challenge inappropriate prescribing by their colleagues (p = 0.001) and were more aware of the importance of antimicrobial stewardship (p = 0.005). Reduced use of the app was associated with agreement that senior physicians’ preferences for antimicrobial prescribing would irrespectively overrule guideline recommendations (p = 0.0002).

**Conclusions:**

Smartphone apps are an effective and acceptable format to deliver guidance on antimicrobial prescribing. Our findings suggest that they may empower users to challenge incorrect prescribing, breaking well-established behaviours, and thus supporting vital stewardship efforts in an era of increased antimicrobial resistance. Future work will need to focus on the direct impact on drug prescriptions as well as identifying barriers to implementing smartphone apps in other clinical settings.

## Introduction

Antimicrobial stewardship is one of the key pillars of the UK’s 5 year strategy against the global spread of antimicrobial resistance [1], aiming to reduce inappropriate prescribing of antimicrobial drugs, thereby reducing selective pressure for resistant organisms. One of the cornerstones of stewardship is appropriate empirical selection of antimicrobial drugs when a treatable infection is clinically suspected (“Start SMART”) [2]. Making an informed decision at the outset is particularly important as the majority of antimicrobial prescriptions on admission remain unchanged [3].

Traditionally, junior doctors in hospitals have been issued with printed pocket guides to antimicrobial prescribing, steering drug choices towards those agreed by the hospital’s antimicrobial usage committee. The success of this approach faces several challenges, such as ensuring doctors carry the pocket guide and the need to issue new physical copies of the guidelines when they are updated. Antimicrobial prescribing guidelines are also commonly available on hospital intranet websites, but hospital IT systems may remain focused on desktop computers, potentially restricting the access and utility of this source of guidance at the patient bedside. These approaches to disseminating hospital guidelines on antimicrobial prescribing have persisted despite evidence demonstrating that antimicrobial prescribing choices are most influenced by peers and more senior physicians. This creates a prescribing culture of accepted non-compliance to policies and non-interference with the antimicrobial drug decisions of colleagues, resulting in reluctance to interfere in prescriptions started by others [4,5].

The recent upturn in smartphone use in the general population has been matched by increased development of smartphone apps geared towards use in healthcare, including in the field of antimicrobial stewardship [6–8]. Clinicians are likely to be in constant contact with their phone, in contrast to pocket guides, desktop computers and reference handbooks. Furthermore, information found on a smartphone might be accessed more frequently at the patient bedside, and be easier to update remotely without needing to issue new physical copies. Despite these theoretical advantages, the acceptance and impact of smartphone apps on prescribing behaviour has not been studied in detail. In August 2013 our hospital replaced a paper-based pocket guide to antimicrobial prescribing with the MicroGuide smartphone app [8]. We took advantage of this to perform a cross-sectional before and after study, hypothesising that the app would improve access to hospital antimicrobial guidelines, and that the ease of access of this information would disrupt conventional prescribing practices.

## Methods

### The smartphone app

MicroGuide (http://www.microguide.eu) is a cloud-based service that allows local pharmacists to create, edit, manage and publish their own clinical guidance to a series of apps for any iOS (Apple, USA), Android (Google, USA) and Windows Phone (WP) device (Microsoft, USA) [WP version not available at the time of the study]. Clinicians and pharmacists have access to a content management portal locally where guidance is created and managed autonomously. The app content was near-identical to the pocket guide, with empiric guidelines for body system infections, sepsis and healthcare associated infections, as well as a dedicated antibiotic stewardship section. A content list of all the app sections is available in S4 File. Download and usage statistics were derived from routine app data collection by Horizon Strategic Partners.

### Study design

The study was performed at University College London Hospitals NHS Trust, a tertiary-care university affiliated NHS Trust with 1093 beds, medically staffed by approximately 700 consultants and 700 sub-consultant grade doctors. In this setting antimicrobial prescribing is performed solely by physicians at all levels of training across all specialties. We conducted two structured cross-sectional surveys that were distributed at the end of July 2013 and November 2013 respectively. These dates corresponded with questionnaire distribution before the release of the smartphone app ("pre-app questionnaire") and several months after the app was established in clinical practice in the hospital ("post-app questionnaire") [S1 and S2 Files]. Both asked the same questions, except that the latter included questions relating to frequency of use of the app and its impact on clinical practice and prescribing. Validity of questionnaire contents was determined using themes previously established in related literature on smartphone app use [9,10]. Some questions, such as open-ended commenting questions, were inserted to provide local feedback about implementation of the app, and were not included in the dataset analysed to derive our findings (S3 File). The pre-app questionnaire was initially piloted amongst an unselected group of 6 junior doctors. Feedback resulted in minor phraseology modifications and these individuals’ responses were excluded from further analyses.

All questionnaire forms were completed anonymously and no personal identifiable information was recorded. Electronic transcribing of paper questionnaire responses was performed by a different person to the one distributing questionnaires, providing an extra layer of anonymity and confidentiality for responders. The anonymised paper forms were then stored in a locked filing cabinet in the Department of Pharmacy during and after the study.

The study focused solely on inpatient antimicrobial prescriptions and thus both questionnaires were distributed in paper format around the hospital over a 2 week period in the same systematic manner, covering all wards and sites of the hospital that offered inpatient care during daytime working hours. Questionnaires were distributed in person by study team members to all medical staff in attendance on the ward during a one hour window. Questionnaire forms taken by a ward physician and returned to study staff formed the basis of the response rate denominators and nominators respectively. Pre- and post-app questionnaire distribution dates straddled the traditional junior doctor rotation day in early August, the same date that had also been pre-determined to release the smartphone app. Consequently, we anticipated there would be minimal overlap in responders between the 2 questionnaires. Verbal enquiry of all post-app responders prior to questionnaire completion confirmed that there was no overlap in respondents between the pre-app and post-app questionnaires. Furthermore, no individual completed either questionnaire more than once. Electronically transcribed data were analysed using Prism (Graphpad Software Inc, USA) and Stata (StataCorp LP, USA).

### Data analysis

Background information about the responder populations was derived from 116 completed pre-app and 146 completed post-app questionnaires. All questionnaire responses used for the analyses presented in this manuscript are available in S3 File. Likert scale responses to the question “I am concerned about the emergence of drug-resistant infections” were quantified on a 5 point scale, and were assessed for differences between the two populations by Mann-Whitney test. Responses relating to frequency of use of a source of information were also derived from an ordinal Likert scale and were converted to a 5 point scale, permitting assessment between frequencies of use of one source of information versus another by Mann-Whitney tests. The frequency scale for smartphone app use was entered into a linear regression model with the year of graduation from medical school, as well as with ordinal Likert agreement responses to questions relating to views about the smartphone app (usefulness, ease of navigation, relevance to patients and ease of access to hospital guidelines).

To assess the relationship between statements relating to the smartphone app and the frequency of its use, we used an unadjusted logistic regression model. Ordinal Likert scales were converted into binary responses: use frequency of the smartphone app was assigned to either ‘high use’, defined by at least daily use or ‘low use’, defined by less frequent than daily use. Agreement statement responses were assigned to either agreement (“Agree” & “Strongly agree” answers) or any other agreement response. The output of the logistic regression model was a risk ratio with confidence interval (C.I.). A risk ratio >1 indicated a positive relationship between agreement to the statement and high use of the smartphone app.

## Results

### Smartphone app implementation

The MicroGuide smartphone app was introduced in our hospital in August 2013, and in the first 10 months, 849 users registered to use the app, and it was downloaded 2013 times. The app was used extensively, being accessed >16 000 times, with an average of 1182 monthly accessions, a figure that remained stable over the first year of use (range 1005–1615 app accessions / month). The subsequent 19 months confirmed this continuous usage with an average of 1483 monthly accessions (range 945–2140 app accessions / month). During the first year of usage, information contained within each individual guideline was accessed for 12.5 seconds on average. This figure did not include time taken to open and navigate the app to each guideline. The accession time fell slightly during the subsequent 19 months of use (10.6 seconds / guideline), possibly reflecting increased familiarity with the app guideline content. The 5 most frequently accessed app sections were (in order) UTI (lower), pneumonia, cellulitis, UTI (upper/pyelonephritis) and sepsis.

### Responder information

We received 116 responses from 149 distributed pre-app questionnaires (response rate 78%) and 146 responses from 185 distributed post-app questionnaires (response rate 79%). Background information about respondents is presented in Table 1, demonstrating that both questionnaires sampled a wide cross-section of doctors within the hospital across a range of specialties with similar levels of expertise, and that the two populations were comparable (Table 1). There was no difference between the populations in the reported frequency they used mobile phones, hospital computers or clinical pocket books to access any medical information (S1 Fig). High levels of concern about antimicrobial resistance was noted in both questionnaire populations (>80%), and did not differ between them (p = 0.81 by Mann-Whitney test) (Table 1). Furthermore, >92% of respondents to both questionnaires regularly carried phones compatible with the smartphone app (Apple & Android), indicating that the smartphone app had the potential to impact the vast majority of the questionnaire respondents.

**Table 1:** Background information on both questionnaire respondent populations.

	Pre-app questionnaire	Post-app questionnaire
**Responders / recipients of questionnaire (response rate %)**	116/149 (78%)	146/185 (79%)
**Specialty**		
*Medicine*	52 (45%)	66 (45%)
*Surgery*	34 (29%)	44 (30%)
*Obstetrics & gynaecology*	5 (4%)	22 (15%)
*Paediatrics*	11 (10%)	8 (6%)
*Others / Not specified*	14 (12%)	6 (4%)
**Grade of respondent**		
*Foundation doctor (within 2 years of graduation)*	24 (21%)	22 (15%)
*Core trainee (2–5 years since graduation)*	47 (40%)	51 (35%)
*SpR+ (5+ years since graduation)*	45 (39%)	73 (50%)
**Carrying a smartphone (%)**	113/116 (97%)	139/146 (95%)
**Smartphone operating system (% of all smartphone users)**		
*Apple*	81 (72%)	97 (70%)
*Android*	26 (23%)	38 (27%)
*Not Apple or Android smartphone*	6 (5%)	4 (3%)
**Concerned about the emergence of drug resistant infections**		
*Strongly disagree*	2 (2%)	2 (1%)
*Disagree*	4 (3%)	6 (4%)
*Neither agree nor disagree*	10 (9%)	18 (12%)
*Agree*	63 (54%)	71 (49%)
*Strongly agree*	37 (32%)	48 (33%)

### Use of the app

For responses to use of the app, we included only the 112 respondents from the post-app questionnaire that were both aware of the smartphone app and who had a compatible smartphone to use it. We initially assessed the relationship between respondents’ year of medical graduation and frequency of app use, and a linear regression model demonstrated that it was the more junior doctors who used the app most frequently (r^2^ = 0.19, p<0.0001).

**Fig 1:**
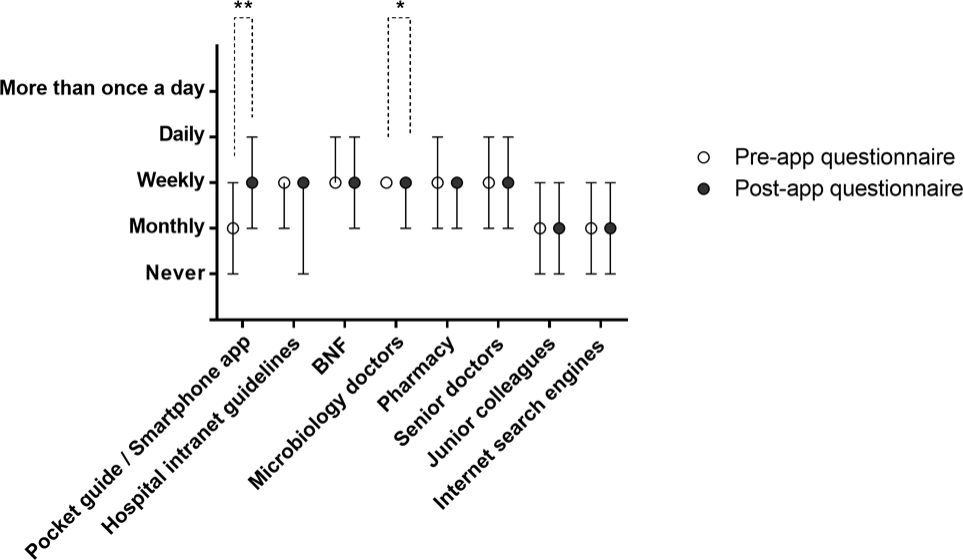
Use frequency of antimicrobial prescribing guidelines before and after introduction of the smartphone app. In the first group, pre-app questionnaire data relate to frequency of use of the pocket guide, whereas post-app questionnaire data relate to frequency of use of the smartphone app. Each data point denotes median frequency of responses and error bars represent interquartile range of frequency of responses. **—denotes smartphone app used more frequently than pocket guide (p < 0.01 by Mann-Whitney test). *—denotes microbiology doctors’ advice used less frequently after introduction of the smartphone app (p < 0.05 by Mann-Whitney test).

To investigate whether the smartphone app was used more frequently than the pocket guide that preceded it, we compared responses from the pre-app questionnaire (n = 116) with the 112 respondents from the post-app questionnaire. For this analysis, questionnaire-reported use of the app was compared directly to reported use of the pocket guide, demonstrating that the app was used significantly more frequently than the pocket guide (p<0.01 by Mann-Whitney test, sum of ranks 11399 for smartphone app vs 14253 for pocket guide) (Fig 1). Concurrently, following introduction of the app, advice from microbiology doctors was used less frequently (p<0.05 by Mann-Whitney test, sum of ranks 11945 for microbiology advice before introduction of the app vs 13707 for microbiology advice after introduction of the app). No difference was seen between the two sets of responders when comparing the other forms of prescribing guidance (Fig 1 and S1 and S2 Files).

### App acceptance and impact on prescribing behaviour

More than 90% of respondents agreed or strongly agreed that the app was useful, easy to navigate, relevant to their patients and the best way to access the hospital guidelines. Agreement with all these questions was strongly related with increased frequency of app use using a linear regression model (r^2^ for each of these variables was 0.295, 0.214, 0.203 and 0.154 respectively. p<0.001 for all).

High use of the app was associated with improved awareness of the importance of antimicrobial stewardship (risk ratio 6.8, C.I. 2.1–21.7), and with agreement that the app encouraged users to challenge inappropriate prescribing they observed in other doctors (risk ratio 3.8, C.I. 1.5–9.7). However, there was no relationship between frequency of use of the app and the sentiment that the app impacted the way antimicrobials were documented on a drug chart (Table 2).

**Table 2:** Relationship between frequency of use of the smartphone app and the perception of the app and barriers to its use.

Agreement statement from post-app questionnaire	Respondents to statement (n)	Risk ratio	C.I.	p value
Increased awareness of antimicrobial stewardship	91	6.8	2.1–21.7	**0.001**
Encouraged to challenge inappropriate prescribing of others	92	3.8	1.5–9.7	**0.005**
Encouraged to document indication for antimicrobials on the drug chart	92	2.2	0.9–5.5	0.07
Encouraged to document duration of antimicrobials on the drug chart	91	1.7	0.7–4.2	0.2
Feeling comfortable using a smartphone on a ward round	90	1.8	0.7–4.8	0.21
Feeling comfortable accessing the app at patient’s bedside	90	1.4	0.59–3.5	0.43
^Δ^ Seniors’ preferences guide antimicrobial prescribing more than hospital guidelines	106	0.03	0.0018–0.5	**0.0002**

Results were derived from the 112 respondents of the post-app questionnaire who declared being aware of the presence of the smartphone app and possessed a compatible smartphone. Statistical analysis by logistic regression. Risk ratio >1 indicated a positive relationship between agreement to the statement and increased use of the smartphone app.

Δ –in this hospital setting, “seniors” relates to physicians practicing after the end of their specialty training (i.e. consultant level).

### Barriers to using the app

The post-app questionnaire also sought to address some of the barriers to using the app in the hospital. App use was not associated with concerns around the use of phones on ward rounds or at the bedside, but reduced use of the app was associated with agreement that senior physicians' preferences guided antimicrobial prescribing more than hospital guidelines (risk ratio 0.03, C.I. 0.0018–0.5) (Table 2).

## Discussion

Our hospital introduced a smartphone app that contains local guidance on antimicrobial prescribing, replacing a paper-based pocket guide, and we used this opportunity to investigate how the app impacted on attitudes and behaviours to antimicrobial prescribing. The smartphone app was well received, and nearly all respondents carried a compatible smartphone. The vast majority used the app at least weekly, and it was used more frequently than the paper based pocket guide that had offered near-identical content. Crucially, the more the app was perceived to be of high quality and utility, the more it was used.

A key and novel finding was that users with high app usage were more likely to feel empowered to challenge inappropriate prescribing by their colleagues. High app use was also associated with users reporting increased awareness of antimicrobial stewardship efforts. Hospitals issue many guidelines to aid their prescribing, but a challenge remains accessibility at the point of need, persuading users of their virtues, as well as the ability of any new form of guidance to overcome common “social norms” seen in antimicrobial prescribing [4]. It is in this context that it is particularly noteworthy that app usage appeared to break the established hierarchical decision making in prescribing antimicrobials, providing an evidence-based incentive to introduce well-designed smartphone apps in other areas of clinical practice [11].

The second notable result was that a key barrier to implementing guidelines on the app remains senior physicians’ preferences for antimicrobial choices. Such a hurdle has previously been elucidated from qualitative studies [7,9], and needs to be considered when introducing smartphone apps in other clinical areas. Potential benefits of using an app need to be explained not just to end-users, but also to individuals whose decision making may be challenged by the content of apps.

It is also noteworthy that not all previously identified obstacles were borne out from our study, such as concerns about using a smartphone in a ward-based setting, which has been perceived as unprofessional by users in other studies [7,9]. Notwithstanding methodological differences, the discrepancy may reflect increased social acceptance of utilising mobile technology in day to day clinical practice by both staff and patients [12].

### Strengths and limitations of the study

To our knowledge, this is the first study that specifically addresses how a smartphone app affects users’ behaviours to antimicrobial prescribing. The study was performed in a routine clinical setting and collected information from a large sample. As expected, the app was used by the more junior members of the hospital, confirming that respondents were representative of app users in our hospital. The nature of the questionnaire study design meant that we could not make causative conclusions about relationships uncovered between variables. However, this limitation does not detract from the novelty of the associations found, as these can now form the basis for testable hypotheses in future studies.

It was not possible to reliably determine the absolute denominator of doctors in work on the days the questionnaires were distributed, but we attempted to minimise any unintentional selection bias in several ways. We maintained a consistent sampling method for both questionnaires and post-app questionnaires were distributed more than 3 months into the start of clinical rotations, equilibrating familiarity with the routine clinical work in both responder populations. In addition, both populations were equally concerned about antimicrobial resistance, and no differences were seen between the cohorts in the frequency they used mobile phones, hospital computers or pocket guides to access any medical information, negating a general effect on respondents’ use of media formats in the clinical setting, although we cannot exclude that another untested variable could have confounded the responses between the two populations.

Finally, although not all questionnaires were completed fully, more than 85% of non-responses to questions about the impact of the smartphone app were found in individuals who had stated they never used the app, affirming the validity of completed responses from regular app users.

## Conclusions

The increased prevalence in antimicrobial resistance obligates prudent prescribing of currently available drugs. This form of stewardship is beginning to harness the utility and ubiquity of smartphones through the use of apps. Our study provides an insight into the impact of this nascent area, demonstrating that a well-designed and accepted smartphone app can increase awareness of the importance of antimicrobial stewardship and influence some prescribing behaviours. In particular, it may break the etiquette of collusion in prescribing, empowering users to challenge others’ incorrect prescribing decisions. Future work will need to directly assess how the app influences the choice of drugs prescribed and the duration of prescriptions. In turn, this would provide more direct evidence that the app challenges established prescribing patterns, as well as aid further exploration of the barriers to app use at both individual and institutional levels.

## Supporting Information

S1 Fig Use frequency of antimicrobial prescribing guidelines before and after introduction of the smartphone app. (TIF)

S1 File Pre-app questionnaire. (DOCX)

S2 File Post-app questionnaire. (DOCX)

S3 File Questionnaire responses. This file contains all the questionnaire responses used to derive the analyses presented in this manuscript.
(XLSX)

S4 File Description of MicroGuide app content. (XLSX)
