# Supplementary figures and images for: Attitudes and Behaviours to Antimicrobial Prescribing following Introduction of a Smartphone App
Source: PLoS One. 2016 Apr 25;11(4):e0154202. doi: 10.1371/journal.pone.0154202 (PMC4844117; doi:10.1371/journal.pone.0154202)

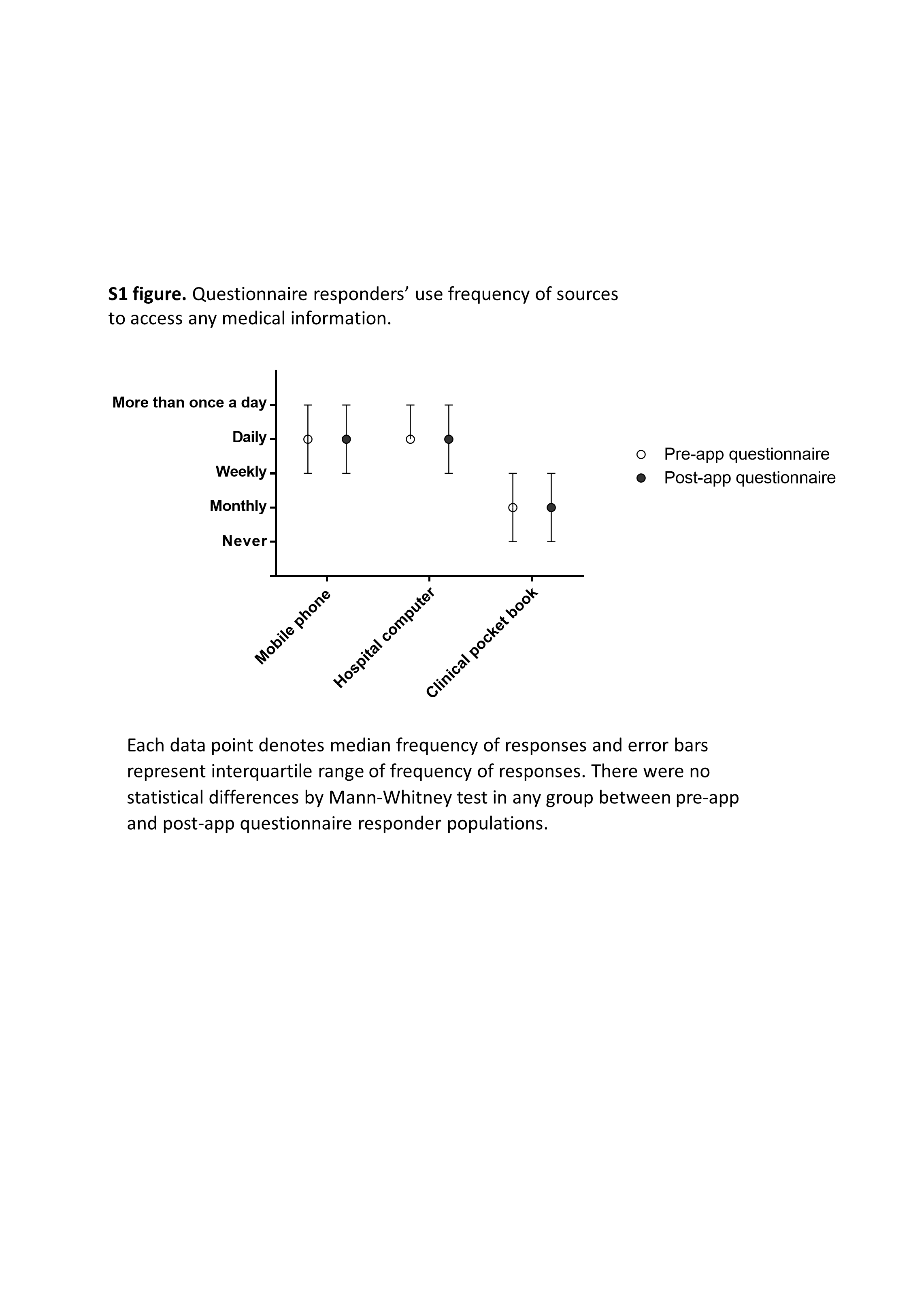

Supplement: S1 Fig — (TIF) [file pone.0154202.s001.tif]
