# Supplementary material for: Attitudes and Behaviours to Antimicrobial Prescribing following Introduction of a Smartphone App
Source: PLoS One. 2016 Apr 25;11(4):e0154202. doi: 10.1371/journal.pone.0154202 (PMC4844117; doi:10.1371/journal.pone.0154202)
Supplement: S1 File — (DOCX) [file pone.0154202.s002.docx]

# **Survey on antimicrobial prescribing practice at UCLH**

**1) Background information**

| Grade |  |
| --- | --- |
| Specialty |  |
| Year of graduation from medical school |  |

|  | More than once a day | Daily | Weekly | Monthly | Never |
| --- | --- | --- | --- | --- | --- |
| **2) How frequently do you prescribe antimicrobials?** | □ | □ | □ | □ | □ |

| **3) How frequently do you access the following sources of information on antimicrobial prescribing?** | | | | | |
| --- | --- | --- | --- | --- | --- |
|  | More than once a day | Daily | Weekly | Monthly | Never |
| a) UCLH Inform guidelines | □ | □ | □ | □ | □ |
| b) UCLH Antibiotic Pocket guide | □ | □ | □ | □ | □ |
| c) BNF / National guidelines | □ | □ | □ | □ | □ |
| d) Microbiology / Infectious Diseases advice | □ | □ | □ | □ | □ |
| e) Pharmacists | □ | □ | □ | □ | □ |
| f) Senior colleagues | □ | □ | □ | □ | □ |
| g) Other junior doctors | □ | □ | □ | □ | □ |
| h) Internet search engines (e.g. Google) | □ | □ | □ | □ | □ |
| i) Other (please state)  ……………………………………………………………. | □ | □ | □ | □ | □ |

**4) Do you carry a smartphone with you at work?**

| Yes | No |
| --- | --- |

**5) If you carry a smartphone, which operating system does it run on?**

| iPhone | Android | Windows Phone | Blackberry | Other (please state) |
| --- | --- | --- | --- | --- |

*Please turn the page to complete the questionnaire*

| **6) How frequently do you access ANY FORM of medical information using the following:** | | | | | |
| --- | --- | --- | --- | --- | --- |
|  | More than once a day | Daily | Weekly | Monthly | Never |
| a) a mobile phone | □ | □ | □ | □ | □ |
| b) a tablet | □ | □ | □ | □ | □ |
| c) a UCLH trust computer | □ | □ | □ | □ | □ |
| d) a pocket book (e.g. Oxford Handbook) | □ | □ | □ | □ | □ |
| e) printed posters | □ | □ | □ | □ | □ |
| f) Other (please state)  ………………………………………………. | □ | □ | □ | □ | □ |

| **7) Please tick the most appropriate answers to the statements below** | | | | | |
| --- | --- | --- | --- | --- | --- |
|  | Strongly disagree | Disagree | Neither agree nor disagree | Agree | Strongly agree |
| a) UCLH Inform antimicrobial guidelines are easy to access | □ | □ | □ | □ | □ |
| b) I usually carry the UCLH Antibiotic Pocket guide with me | □ | □ | □ | □ | □ |
| c) UCLH antimicrobial guidelines don’t apply to my patients | □ | □ | □ | □ | □ |
| d) My seniors' preferences guide antimicrobial prescribing more than UCLH guidelines | □ | □ | □ | □ | □ |
| e) I prefer to use non-UCLH guidelines to guide my antimicrobial prescribing | □ | □ | □ | □ | □ |
| f) I am concerned about the emergence of drug-resistant infections | □ | □ | □ | □ | □ |

*Thank you for taking the time to complete the questionnaire*
