# Supplementary material for: Attitudes and Behaviours to Antimicrobial Prescribing following Introduction of a Smartphone App
Source: PLoS One. 2016 Apr 25;11(4):e0154202. doi: 10.1371/journal.pone.0154202 (PMC4844117; doi:10.1371/journal.pone.0154202)
Supplement: S2 File — (DOCX) [file pone.0154202.s003.docx]

# **Survey on antimicrobial prescribing practice at UCLH**

**1) Background information**

| Grade | ……………………………... | Specialty | ……………………….……. | Year of graduation  from medical school | ………………….…………. |
| --- | --- | --- | --- | --- | --- |

| **2) How frequently do you access ANY FORM OF MEDICAL INFORMATION using the following:** | | | | | |
| --- | --- | --- | --- | --- | --- |
|  | More than once a day | Daily | Weekly | Monthly | Never |
| a mobile phone | □ | □ | □ | □ | □ |
| a tablet | □ | □ | □ | □ | □ |
| a UCLH trust computer | □ | □ | □ | □ | □ |
| a pocket book (e.g. Oxford Handbook) | □ | □ | □ | □ | □ |
| printed posters | □ | □ | □ | □ | □ |
| Other (please state)  ………………………………………………. | □ | □ | □ | □ | □ |

|  | More than once a day | Daily | Weekly | Monthly | Never |
| --- | --- | --- | --- | --- | --- |
| **3) How frequently do you prescribe antimicrobials?** | □ | □ | □ | □ | □ |

| **4) Please tick the most appropriate answers to the statements below** | | | | | |
| --- | --- | --- | --- | --- | --- |
|  | Strongly disagree | Disagree | Neither agree nor disagree | Agree | Strongly agree |
| UCLH **Inform** antimicrobial guidelines are easy to access | □ | □ | □ | □ | □ |
| My seniors' preferences guide antimicrobial prescribing more than UCLH guidelines | □ | □ | □ | □ | □ |
| UCLH antimicrobial guidelines don’t apply to my patients | □ | □ | □ | □ | □ |
| I prefer to use non-UCLH guidelines to guide my antimicrobial prescribing | □ | □ | □ | □ | □ |
| I am concerned about the emergence of drug-resistant infections | □ | □ | □ | □ | □ |

**5) Do you carry a smartphone with you at work?**

| Yes | No |
| --- | --- |

**6) Which operating system does it run on?**

| iPhone | Android | Windows Phone | Blackberry | Other (please state)  ………………………………….. |
| --- | --- | --- | --- | --- |

**7) Are you aware that UCLH has an Antimicrobial Smartphone App available?**

| Yes | No |
| --- | --- |

| **8) How frequently do you access the following sources of information on ANTIMICROBIAL PRESCRIBING?** | | | | | |
| --- | --- | --- | --- | --- | --- |
|  | More than once a day | Daily | Weekly | Monthly | Never |
| UCLH Antimicrobial App | □ | □ | □ | □ | □ |
| UCLH Inform guidelines | □ | □ | □ | □ | □ |
| BNF / National guidelines | □ | □ | □ | □ | □ |
| Microbiology / Infectious Diseases advice | □ | □ | □ | □ | □ |
| Pharmacists | □ | □ | □ | □ | □ |
| Senior colleagues | □ | □ | □ | □ | □ |
| Other junior doctors | □ | □ | □ | □ | □ |
| Internet search engines (e.g. Google) | □ | □ | □ | □ | □ |
| Other (please state)  ……………………………………………………………. | □ | □ | □ | □ | □ |

| **9) Please tick the most appropriate answers to the statements below about THE UCLH ANTIMICROBIAL SMART PHONE APP** | | | | | |
| --- | --- | --- | --- | --- | --- |
|  | Strongly disagree | Disagree | Neither agree nor disagree | Agree | Strongly agree |
| I have found the app useful | □ | □ | □ | □ | □ |
| The app is easy to navigate | □ | □ | □ | □ | □ |
| The content of the app is relevant to my ­patient population | □ | □ | □ | □ | □ |
| The app is the best way to access UCLH antimicrobial guidelines | □ | □ | □ | □ | □ |
| I would like to see all UCLH antimicrobial guidelines on the app | □ | □ | □ | □ | □ |
| The app encourages me to document the **indication** for antimicrobials on the drug chart | □ | □ | □ | □ | □ |
| The app encourages me to document the **duration** for antimicrobials on the drug chart | □ | □ | □ | □ | □ |
| The app encourages me to challenge inappropriate prescribing by others | □ | □ | □ | □ | □ |
| I feel comfortable using a smart phone on a ward round | □ | □ | □ | □ | □ |
| I feel comfortable accessing the app at the patient’s bedside | □ | □ | □ | □ | □ |
| The app has increased my awareness of antimicrobial stewardship | □ | □ | □ | □ | □ |

| **10) Do you have any suggestions for improving the content of the UCLH Antimicrobial smart phone app?** |  |
| --- | --- |
| **11) Please detail any other comments you have about the UCLH antimicrobial smart phone app** |  |
| **12) How many other medical apps do you use on a regular basis? Please state their names.** |  |
